# The Incidence and Treatment Response of Double Expression of MYC and BCL2 in Patients with Diffuse Large B-Cell Lymphoma: A Systematic Review and Meta-Analysis

**DOI:** 10.3390/cancers13133369

**Published:** 2021-07-05

**Authors:** Jisun Hwang, Chonghyun Suh, Kyungwon Kim, Hosung Kim, Austin I. Kim, Jeffrey W. Craig, Ke Xun Chen, Joel Roberson, Jeffrey P. Guenette, Raymond Y. Huang

**Affiliations:** 1 Department of Radiology, Dongtan Sacred Heart Hospital, Hallym University Medical Center, 7, Keunjaebong-gil, Hwaseong-si 18450, Gyeonggi-do, Korea; biydjs@hallym.or.kr; 2 Department of Radiology and Research Institute of Radiology, University of Ulsan College of Medicine, Asan Medical Center, Olympic-ro 33, Seoul 05505, Korea; kyungwon_kim@amc.seoul.kr (K.K.); radhskim@gmail.com (H.K.); 3 Center for Hematologic Oncology, Dana-Farber Cancer Institute, Harvard Medical School, Boston, MA 02115, USA; AustinI_Kim@dfci.harvard.edu; 4 Centre for Lymphoid Cancer, British Columbia Cancer, Vancouver, BC V5Z 4E6, Canada; jeffrey.craig@bccancer.bc.ca; 5 Division of Neuroradiology, Brigham and Women’s Hospital, Dana-Farber Cancer Institute, Harvard Medical School, 75 Francis Street, Boston, MA 02115, USA; kexunchen@gmail.com (K.X.C.); Joel2r3@gmail.com (J.R.); JPGUENETTE@bwh.harvard.edu (J.P.G.); ryhuang@bwh.harvard.edu (R.Y.H.)

**Keywords:** meta-analysis, systematic review, lymphoma, immunohistochemistry

## Abstract

**Simple Summary:**

Diffuse large B-cell lymphoma (DLBCL) with co-expression of MYC and BCL2 proteins is referred to as double expressor lymphoma. Multiple studies have identified double expressor status to be an adverse predictive factor for response to standard chemotherapy regimens. The revised 2016 WHO classification recommends cutoff values of 40% for MYC and 50% for BCL2 protein expression; however, actual cutoff values have varied widely among published studies. Increasing recognition of the potential prognostic value of double expressor status prompted this systematic review and meta-analysis of the worldwide literature. Our findings indicate that approximately 23% of de novo DLBCL tumors express both MYC and BCL2 proteins above the indicated thresholds. Remarkably, different immunohistochemical cutoff values did not significantly affect the proportion of tumors attaining double expressor status. Cases lacking MYC/BCL2 co-expression were associated with a significantly higher probability of complete remission, thereby reaffirming the value of this predictive biomarker.

**Abstract:**

MYC/BCL2 protein co-expression (i.e., double expressor) has been shown to be a negative predictor of outcome in diffuse large B-cell lymphoma (DLBCL). We aimed to establish the incidence of double expressor status in patients with de novo DLBCL and identify the predictive value of this biomarker on treatment response through systematic review and meta-analysis. PubMed and Embase were searched for studies published through December 2019 that reported proportions of double expressor DLBCL. The pooled proportions of MYC and BCL2 expression, both alone and in combination, were computed using the inverse variance method for calculating weights and by the DerSimonian–Laird method. The pooled odds ratios (ORs) of complete remission (CR) rate were calculated, and meta-regression analysis was conducted to explore heterogeneity. Forty-one studies (7054 patients) were included. The pooled incidence of double expressor status in DLBCL was 23% (95% confidence interval [CI], 20–26%), with an adjusted estimate of 31% (95% CI, 27–36%). Neither MYC/BCL2 protein cutoff values, race, mean, or median age of included patients, or overall study quality was a significant factor of heterogeneity (*p* ≥ 0.20). Cases without double expressor status demonstrated a higher probability of CR to rituximab, cyclophosphamide, doxorubicin, vincristine, and prednisone treatment (OR, 2.69; 95% CI, 1.55–4.67). Our results reaffirm the predictive power of this important biomarker.

## 1. Introduction

Diffuse large B-cell lymphoma (DLBCL) is the most common subtype of non-Hodgkin lymphoma [1]. The standard R-CHOP (rituximab, cyclophosphamide, doxorubicin, vincristine, and prednisone) chemotherapy regimen results in cure in up to 60% of patients [2]. A vigorous search has been made for biomarkers that can predict patients at high risk for treatment failure. Clinical and molecular factors including age, International Prognostic Index (IPI) score, molecular cell-of-origin (COO), chromosomal rearrangements, and protein expression have been identified as potential prognostic factors [3,4,5,6]. High-grade B-cell lymphoma with *MYC* and *BCL2* and/or *BCL6* rearrangements, so called double-hit and triple-hit (DH/TH) lymphomas, are defined in the 2016 World Health Organization (WHO) classification as a new diagnostic category and includes a subset of tumors with DLBCL morphology [7]. The concept of atypical DH lymphoma has also been proposed for cases harboring copy number variations in both *MYC* and/or *BCL2* in the absence of concurrent translocations [8]. However, recent evidence shows that *MYC* and *BCL2* copy number variations do not produce the high-risk gene expression signature seen in most true DH/TH lymphomas harboring *MYC* and *BCL2* rearrangements, suggesting that copy number variations should not be used to expand the definition of DH/TH lymphomas [9].

Expression of MYC and BCL2 proteins is identified by immunohistochemistry (IHC) in some patients with DLBCL even when the chromosomal rearrangements of DH/TH lymphoma are not present. DLBCL tumors that co-express both MYC and BCL2 proteins (regardless of genetic rearrangement) are referred to as double expressor lymphomas. In the absence of chromosomal translocation, elevated protein expression is often mediated through alternative changes such as genetic gains/amplifications or mutations [10]. For example, MYC expression is tightly regulated in normal cells, but becomes dysregulated in up to 70% of all human cancers [11]. The most important mechanisms underlying abnormal MYC protein expression include: (1) structural alterations (e.g., MYC translocation or amplification), (2) enhanced transcription (e.g., super-enhancer activation [12]; PVT1 promoter deletion [13]; and aberrant upstream signaling, particularly B-cell receptor and NF-κB pathways [14]), and (3) altered protein stability (e.g., MYC T58 mutations [15]; and direct phosphorylation by Aurora B Kinase [16]. Multiple studies have identified double expressor status to be an adverse prognostic factor for response to R-CHOP in DLBCL [3,17,18,19]. Concurrent double expressor status has even been associated with poorer outcomes in tumors harboring DH cytogenetics [17,20]. Further, cases with double expressor status have demonstrated distinctive clinical features such as older age and advanced stage [20,21], higher LDH level [22], higher Ki67 proliferation index [23], and higher international prognostic index [24]. The revised 2016 WHO classification recommends cutoff values of 40% for MYC and 50% for BCL2 expression as assessed by immunohistochemistry (IHC) [7]; however, actual cutoff values have varied widely among published studies.

Increasing recognition of the potential prognostic value of MYC and BCL2 co-expression prompted this systematic review and meta-analysis of the worldwide literature. The primary aim of this study was to establish the incidence of double expressor status in patients with de novo DLBCL using pooled estimates according to different IHC cutoff values. The secondary aim of this study was to identify the predictive value of double expressor status on treatment response through meta-analysis.

## 2. Methods

This systematic review and meta-analysis is organized according to the Preferred Reporting Items for Systematic Reviews and Meta-Analyses (PRISMA) statement [25].

### 2.1. Search Strategy and Selection Criteria

PubMed and Embase were searched for articles and abstracts published through 4 December 2019, using the following search terms: ((diffuse large B cell lymphoma) OR (DLBCL)) AND ((double hit) OR (double expressor) OR (dual expressor) OR (myc bcl2) OR (myc bcl-2)). The language was restricted to English.

The inclusion criteria were as follows: (1) patients with newly diagnosed or de novo DLBCL; and (2) detailed data sufficient to assess the proportion of MYC/BCL2 protein co-expression. The exclusion criteria were as follows: (1) conference abstracts, review articles, opinions, letters, comments, editorials, guidelines, case reports, systematic reviews; (2) studies conducted in animals; (3) studies including primary CNS lymphoma; (4) insufficient data for evaluating outcome; and (5) overlapping study populations and data. Studies with larger sample sizes were selected when overlapping with smaller studies. Manual searches (using Google Scholar) for articles describing the use of the DLBCL90 NanoString gene expression assay were conducted to assess the prevalence of the double-hit gene expression signature (DHITsig) and DH/TH lymphoma in germinal center B-cell-like (GCB)-type DLBCL.

### 2.2. Data Extraction and Quality Assessment

The following data were extracted using a standardized data form:

1. Study: authors, publication year, patient enrollment period, institution, country, design.
2. Pathological data: cut-off values of MYC and BCL2 protein expression by IHC, proportion of positive tumor cells for each marker and double expressor status, IHC protocol details.
3. Patient: number of patients, age, gender, clinical setting, international prognostic index, Ann Arbor Stage, prevalence of elevated LDH, treatment arm, complete remission (CR) rate.
4. DHIT-sig: proportion of DHITsig-positive cases, proportion of DH/TH lymphoma, the numbers of true positives, true negatives, false positives, and false negatives of DHIT-sig for predicting DH/TH lymphoma.

The quality of included studies was assessed using the Newcastle-Ottawa scale for cohort and case-control studies [26,27]. The Newcastle-Ottawa scale consists of three domains (Selection, Comparability, and Outcome). A study can be awarded a maximum of one point for each item in the Selection and Outcome domains, and two points for each item in the Comparability domain. The total (sum) of all scores reflects the overall quality of a given study: 8–9, very good; 6–7, good; 4–5, satisfactory; 0–3, unsatisfactory [26]. Data extraction and quality assessment were performed by two independent reviewers (J.H. and C.H.S.) and disagreements were settled by consensus.

### 2.3. Data Synthesis and Analysis

The primary outcome was the pooled proportion of double expressor status among de novo DLBCL tumors. The secondary outcomes were as follows: (1) the results of subgroup analysis for the studies according to cut-off values of MYC and BCL2 protein expression, (2) pooled proportions of MYC and BCL2 protein expression (separately), (3) pooled odds ratio (OR) for CR rate in those with and without MYC/BCL2 protein co-expression.

The pooled proportions of double expressor status as well as MYC and BCL2 protein expression (independently) were computed using the inverse variance method for calculating weights and by the DerSimonian–Laird method [28]. For the analysis of MYC and BCL2 protein expression, the pre-determined cut-off values from individual studies were used. The pooled OR with 95% confidence interval (CI) was calculated with double expressor status as the base category. Pooled estimates of sensitivity and specificity were calculated using a bivariate random effects model [29]. Study heterogeneity was evaluated using the inconsistency index (I^2^) of Higgins et al. [30] with a cut-off of 50%, and the Q test with a *p*-value < 0.10 used to indicate statistical heterogeneity. Data were meta-analytically pooled using a random effects model for more conservative assessment of the incidence of double expressor status and ORs of CR rate [28]. Publication bias was assessed using visual inspection of funnel plots and Eggers test with a value <0.1 used to indicate significant bias [31]. Meta-regression analyses were conducted according to the cut-off values of MYC and BCL2 protein expression, mean or median age of patients, overall study quality, and race. The median age value calculated from the included studies was used as a cut-off for heterogeneity exploration. Statistical analysis was conducted by one author (C.H.S.) with the “meta” and “mada” packages in R software version 3.6.1 (R Foundation for Statistical Computing).

## 3. Results

### 3.1. Literature Search and Quality Assessment

**Figure 1:**
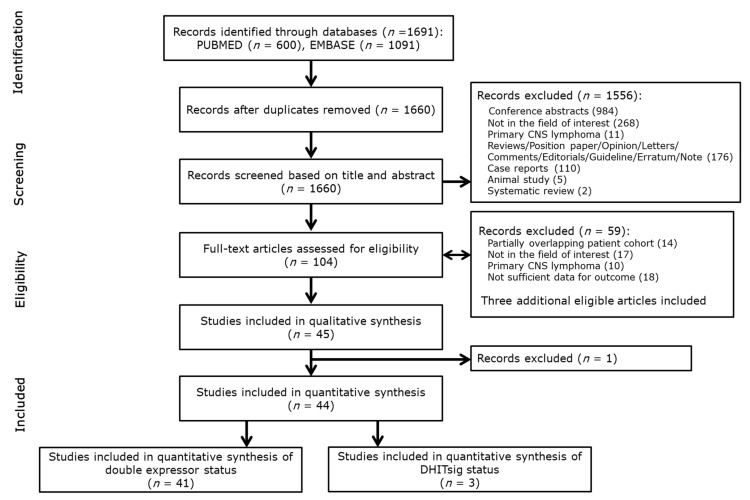
Flow diagram showing study selection process for systematic review.

A total of 1691 articles were initially retrieved by our systematic search. Thirty-four duplicate studies were removed and 1556 articles were further excluded after screening titles and abstracts (Figure 1). After reviewing the full-text of 101 potentially eligible articles, 57 studies were removed due to following reasons: 14 studies included partially overlapping patient cohorts, 17 studies were outside the field of interest, 10 studies reported data on primary CNS lymphoma, and 18 studies lacked necessary outcome data. One additional study was removed after quality assessment. This study, by Wang et al., selected patients based on pre-determined outcomes [32]. In total, 41 studies encompassing 7054 patients were retained for further analysis of double expressor status [3,19,20,21,33,34,35,36,37,38,39,40,41,42,43,44,45,46,47,48,49,50,51,52,53,54,55,56,57,58,59,60,61,62,63,64,65,66,67,68,69].

Since the Newcastle-Ottawa scale was designed for cohort and case-control studies, we considered the selection domains for six secondary analysis studies of previous clinical trials to be of good quality (i.e., four points awarded). Overall, 28 studies received a “very good” quality rating and 13 studies a “good” quality rating (Table S1).

Three additional articles were retrieved due on their inclusion of DLBCL90 NanoString assay data [70,71,72]. All three of these studies received “very good” quality ratings (total scores of nine), although none provided sufficient details regarding the adequacy of follow up (outcome domain).

### 3.2. Characteristics of the Included Studies

The study and patient characteristics of the 41 included studies are listed in Table 1 and Table 2, and Table S2. Detailed antibody information was available in all but one study (40/41, 98%) (Table S3). For the majority of studies (27/41, 66%), additional information regarding the staining platform or other technical conditions was also included. IHC interpretation was performed by hematopathologists or other pathologists nearly three-quarters of the time (30/41, 73.2%).

In brief, the study design was prospective in one study [59], retrospective in 22 [3,32,33,36,38,39,40,42,44,45,46,47,49,50,51,54,60,61,63,66,67,69], secondary analysis of primary clinical trials in six [37,41,48,53,57,58], and not-explained in 12 [19,20,21,34,35,43,52,55,56,62,65,68]. The number of patients per study ranged from 20 to 688, with median ages of 46–70 years. Regarding cutoff values for MYC and BCL2 protein expression, 21 studies used >40% and >50% [20,33,34,35,38,39,41,42,43,44,45,47,49,56,57,58,60,62,63,64,69], 10 studies used >40% and >70% [3,19,48,50,51,53,54,66,67,68], and two studies used >30% and >30% [55,65]. Six studies used other various criteria to define MYC and BCL2 protein expression [21,36,40,46,52,61]. Among the included studies, five evaluated only extra-nodal DLBCL (gastrointestinal, colorectal, and primary bone DLBCL) [38,44,55,59,65]. Sixteen studies were performed in Asian countries [38,42,43,44,45,46,49,54,59,60,61,63,65,67,68,69], 12 in Europe [3,19,34,39,40,48,50,51,53,55,57,58], and six in North America [35,37,52,56,64,66]. The proportions of MYC and BCL2 protein expression were reported in 29 studies [19,20,21,37,38,39,40,42,43,44,45,46,47,48,49,50,51,52,53,54,55,57,58,62,64,65,66,68,69] and 30 studies [19,20,21,33,37,38,39,40,42,43,44,45,46,47,48,49,50,51,52,53,54,55,57,58,62,64,65,66,68,69], respectively.

### 3.3. Meta-Analytic Pooled Prevalence of Double Expressor Status and MYC and BCL2 Protein Expression

**Figure 2:**
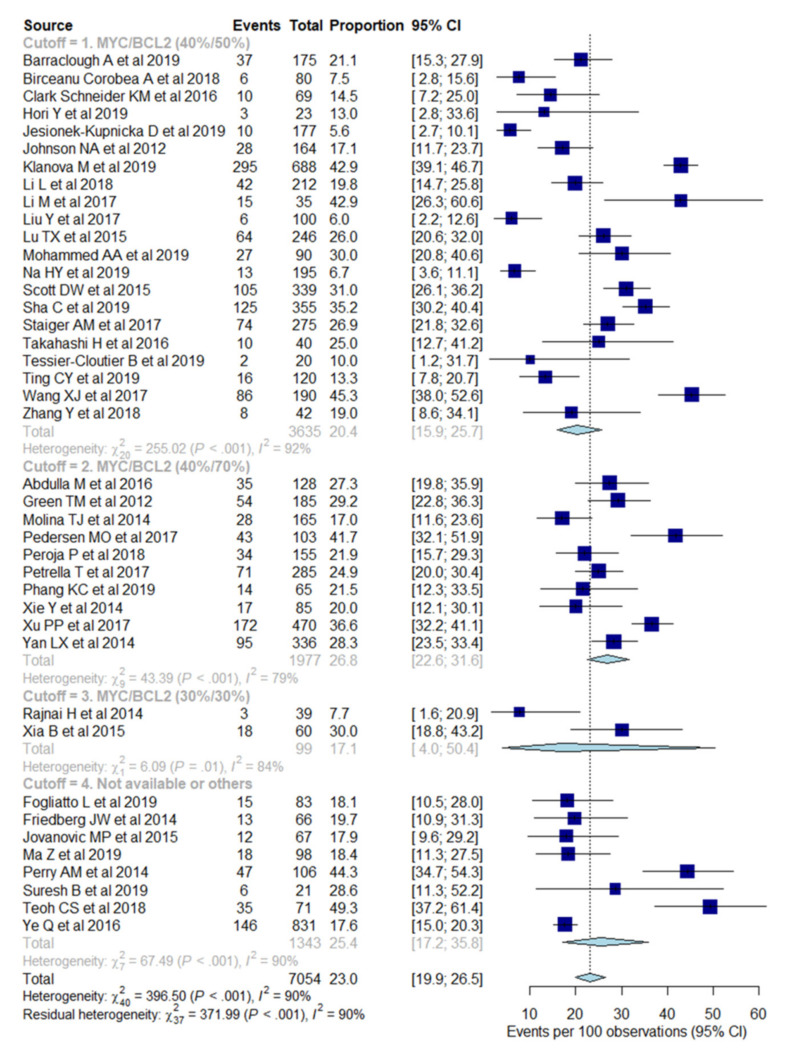
Forest plots show pooled proportion of double protein expression of MYC and BCL2, stratified to different cutoff values by immunohistochemical staining. The events represent double expressor status. The blue box represents the point estimate and its area represents the weight given to the study and a horizontal line indicates the 95% confidence interval. The diamond represents the combined results and the length of the diamond indicates the confidence interval of the pooled results. At the bottom of the plot, the overall pooled proportion is represented by the dashed vertical line and the diamond.

The pooled outcomes for the 41 included studies are summarized in Table 3. The proportion of DLBCL tumors attaining double expressor status varied between 6% and 50%, with a pooled proportion of 23% (95% CI, 20–26%), with significant heterogeneity between studies (I^2^ = 90%, *p* < 0.001) (Figure 2). The funnel plot (Figure S1) and Egger’s test (*p* < 0.001) revealed publication bias. After using the trim-and-fill method, the publication-bias-adjusted pooled proportion was 31% (95% CI, 27–36%).

The pooled proportions of MYC and BCL2 protein expression were 34% (95% CI, 30–39%) and 58% (95% CI, 53–62%), respectively, with significant heterogeneity between studies (all I^2^ > 50%, *p* < 0.001). Egger’s test showed no publication bias in analyses of MYC or BCL2 protein expression (all *p* > 0.1).

### 3.4. Heterogeneity Exploration

Subgroup analysis was performed for the different combinations of cutoff values for MYC and BCL2 protein expression (>40% and >50%, >40% and >70%, >30% and >30%, and not-available, respectively), race, mean, or median age of included patients, and overall study quality (Table 3). The pooled proportion of double expressor status from studies using cut-off values of >40% and >50% was 20% (95% CI, 16–26%), with significant heterogeneity between studies (I^2^ = 92%, *p* < 0.001) (Figure 2). The funnel plot (Supplementary Materials Figure S1) and Egger’s test (*p* < 0.001) showed publication bias, and the publication-bias-adjusted pooled proportion of double expressor status from studies using cutoff values of >40% and >50% was 32% (95% CI, 26–39%). The pooled proportion of double expressor status from studies using cutoff values of >40% and >70% was 27% (95% CI, 23–32%), with significant heterogeneity between studies (I^2^ = 79%, *p* < 0.001) (Figure 2). The funnel plot (Figure S1) and Egger’s test (*p* = 0.07) showed publication bias, and the publication-bias-adjusted pooled proportion of double expressor status from studies using cutoff values of >40% and >70% was 30% (95% CI, 25–35%).

The pooled proportion of double expressor status from studies performed in Asian countries was 23% (95% CI, 17–28%), in Europe was 21% (95% CI, 17–27%), and in North America was 29% (95% CI, 20–39%), with significant heterogeneity between studies (I^2^ > 50%). The pooled proportion of double expressor status in studies of very good quality was 24% (95% CI, 20–28%) and in studies of good quality was 21% (95% CI, 16–28%), with significant heterogeneity between studies (I^2^ > 50%). The pooled proportion of double expressor status in studies with mean or median age ≥60.3 was 25% (95% CI, 19–31%) and in studies with mean or median age <60.3 was 20% (95% CI, 16–24%), with significant heterogeneity between studies (I^2^ > 50%). Upon meta-regression analyses, all covariates (cut-off values for MYC and BCL2 protein expression, race, mean or median age of included patients, and overall study quality) were shown not to be significant factors of heterogeneity with *p*-values of 0.28, 0.56, 0.20, and 0.49, respectively.

### 3.5. Odds Ratio for Complete Remission Rate in Those with and without MYC/BCL2 Protein Co-Expression

**Figure 3:**
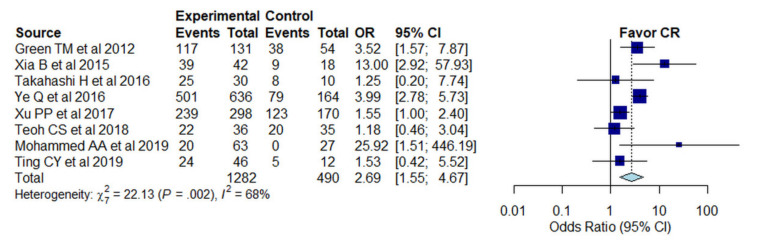
Forest plot shows the pooled odds ratio of complete remission in patients, stratified by double expressor status. The events represent complete remission. The blue box represents the point estimate and its area represents the weight given to the study. Horizontal line indicates the 95% confidence interval. At the bottom of the plot, the overall pooled odds ratio is represented by the dashed vertical line and the diamond. The solid vertical line indicates no effect.

CR rates from subjects with and without MYC/BCL2 protein co-expression were available from eight studies [3,21,47,60,61,63,65,67]. Cases without double expressor status had a significantly higher probability for achievement of CR (combined OR, 2.69; 95% CI, 1.55–4.67) than cases with double expressor status with significant heterogeneity between studies (I^2^ = 68%, *p* < 0.01) (Figure 3). Publication bias could not be assessed due to the small number of included studies.

### 3.6. Evaluation of the Double-Hit Gene Expression Signature in De Novo DLBCL

Study details and patient characteristics are provided in Table S4. Based on a limited number of available studies (*n* = 3), the pooled prevalence of DHITsig was 25% (95% CI, 17.6–35.1; I^2^ = 59.8%) among GCB-type DLBCL. The pooled sensitivity of DHITsig for detecting DH/TH lymphoma was 83.5% (95% CI, 66.6–92.8; I^2^ = 59.8%). The area under the hierarchical summary receiver operating characteristic curve was 0.901. The pooled prevalence of DH/TH lymphoma among DHITsig-positive cases was 48.3% (95% CI, 36.9–59.8; I^2^ = 9.7%). The pooled prevalence of double expressor status among DHITsig-positive cases was 53.6% (95% CI, 41.6–65.2; I^2^ = 33.0%).

## 4. Discussion

In this systematic review and meta-analysis, we established the incidence of MYC/BCL2 co-expression in all de novo DLBCL studies published to date and calculated pooled estimates of double expressor status according to different IHC cutoff values. Our analysis of 41 studies meeting selection criteria revealed that approximately 23% of de novo DLBCL tumors express both MYC and BCL2 proteins. Variably utilized cutoff values for MYC and BCL2 protein expression, mean or median age of included patients, race and overall study quality did not significantly influence the proportion of tumors attaining double expressor status. Pooled estimates confirmed a significantly higher probability of complete remission (OR, 2.69; 95% CI, 1.55–4.67) in cases without double expressor status. This comprehensive review of both the Western and Eastern literature provides a summary of worldwide data relating to double expressor DLBCL and its influence on therapeutic response.

Unlike DH/TH lymphoma, double expressor lymphoma is not regarded as a separate diagnostic entity in the current WHO blue book. Instead, assessment of double expressor status is best viewed as a valuable complement to routine DH/TH fluorescence in situ hybridization (FISH) testing that can help to identify potentially aggressive tumors that are missed by conventional cytogenetic techniques. Indeed, double expressor DLBCL has a much higher reported prevalence of 20–30% [73] compared to the 6–14% prevalence of DH/TH DLBCL [2], and this meta-analysis confirms a pooled proportion of double expressor status of 23% with a publication-bias-adjusted estimate of 31%. Another key feature of double expressor status is that the laboratory infrastructure and immunohistochemical reagents required to perform this testing are widely available due to the important diagnostic roles of both MYC and BCL2 in lymphoma pathology workups. By comparison, FISH analysis is far more costly, time-consuming and requires extra expertise and may therefore not always be available outside of referral laboratories and academic medical centers. Restricting FISH testing to the subset of GCB-type DLBCL with MYC and BCL2 co-expression has been proposed as a cost-effective approach for identifying high-risk patients [14]; however, this strategy would fail to identify roughly one-third of all DH/TH lymphomas [74]. Even when a selective approach to FISH testing is employed, double expressor status should never be viewed as a functional equivalent or alternative to the former. Many double expressor lymphomas do not harbor DH/TH cytogenetics, and the opposite is not always true either. For example, the MYC N11S variant, encoded by a common germline SNP, has been shown to hamper the immunohistochemical detection of MYC [9], and somatic mutations within the *BCL2* gene are a known cause of false negative IHC results [75].

Beyond cell-of-origin, several recent studies have supported the use of gene expression profiling for the prediction of outcome in DLBCL. For example, Sha et al. described a “molecular high-grade” gene expression signature with distinct molecular features and poor outcomes irrespective of DH status [57]. Similar work by Ennishi et al. showed that a “double-hit gene expression signature” (DHITsig; captured by the DLBCL90 NanoString assay) can be used to identify cases of GCB-type DLBCL that share the same aggressive underlying biology exhibited by most GCB-type DLBCL tumors with *MYC* and *BCL2* rearrangements [70]. Based on their data, nearly one-third of GCB-type DLBCL tumors express DHITsig, but only half of those have DH/TH cytogenetics by breakapart FISH or co-express both MYC and BCL2 proteins [70]. Hilton et al. subsequently described a collection of DHITsig-positive tumors lacking DH/TH cytogenetics by breakapart FISH, but in which cryptic *MYC* or *BCL2* rearrangements were detectable by whole-genome sequencing, thus making them true high-grade B-cell lymphomas with *MYC* and *BCL2* rearrangements. Importantly, all of those cases were double protein expressors, highlighting the ability of MYC/BCL2 IHC to catch a few of the aggressive GCB-type DLBCL tumors that are falsely negative by breakapart FISH [76]. Collectively, the studies referenced above indicate that gene expression profiling can identify additional high-risk DLBCL tumors that are missed by FISH and IHC. Unfortunately, gene expression profiling is currently beyond the capabilities of most clinical laboratories and was not used by the vast majority of studies included in this work. In fact, we were only able to identify three published articles reporting the use of the DLBCL90 NanoString assay in DLBCL. Our preliminary assessment of these studies shows consistency across cohorts in regards to the overlap of DHITsig and DH/TH lymphoma. Future meta-analysis research will be needed to understand the broader impacts of novel gene expression signatures once clinical technologies have sufficiently advanced.

The WHO-recommended cutoff values of >40% and >50% were the most commonly used thresholds for defining MYC and BCL2 protein expression, respectively, within published studies meeting our inclusion criteria (21/41, 51%). This was followed by cut-off values of MYC >40% and BCL2 >70% (10/41, 24%). However, these study-specific cutoff values did not significantly influence the proportions of reported double expressor status. Our results might suggest that several of the most commonly used cutoff values are similarly efficacious for defining double protein expression in patients with DLBCL. Alternatively, they would also appear to reflect the subjective nature of IHC assessment as well as other practical limitations discussed below.

Some studies have suggested that racial differences could account for response patterns and survival rates in patients with DLBCL [77,78]. For example, Chen et al. compared 124 Chinese and 114 Western patients with DLBCL and their results suggested that BCL2 expression was more common in Chinese than Western cases [78]. Our meta-analytic results indicate that race is not significant influence on double expressor status.

Double expressor status has repeatedly been shown to be a negative predictor of survival [2,7,73]. Hu et al. reported significantly poorer survival in 893 de novo DLBCL patients with double expressor status treated with R-CHOP with a 5-year overall and progression free survival of <30% [79]. Klanova et al. reported higher CNS relapse rates in patients with dual expressor status in the phase 3 GOYA study, but without statistical significance [41]. A previous meta-analysis revealed that double expressor status was related to poor overall survival in R-CHOP treated DLBCL [80]. In addition to previous studies, our meta-analytic results show that the absence of MYC and BCL2 co-expression is associated with a higher CR rate to R-CHOP (OR, 2.69; 95% CI, 1.55–4.67), thereby reaffirming the clinical significance of this predictive biomarker.

There are several limitations to our work. Significant heterogeneity exists among the analyzed studies. Although we performed subgroup analyses to identify possible sources of heterogeneity, no significant variables were found and thus unidentified causes of heterogeneity are likely to exist. The accurate determination of MYC and BCL2 protein expression can be challenged by numerous obstacles including specimen limitations (e.g., tissue quantity and sample preservation) and technical factors (e.g., IHC reagents and conditions) [81,82], as well as by subjective evaluation. Additionally, the two included studies did not report on the IHC cut-off values for MYC and/or BCL2 protein expression [37,59]. All these might underlie a portion of the unexplained heterogeneity among studies. In the subgroup analysis according to the age of patients, individual patient-level data was not available to perform an appropriate age analysis (i.e., comparing all of the young patients from all of the studies against all of the old patients from all of the studies), and this might explain the non-significant result. Six of eight (75%) studies evaluated for CR were conducted in a retrospective design introducing the possibility that the pooled OR was overestimated. Various cutoff values were used between studies to define double expressor status. Although our results demonstrate that IHC cutoff values did not significantly influence the proportions of double expressor status, the different cutoff values might be a confounding factor for pooled estimates of CR rates. Due to the absence of sufficient data on CR rate in studies that utilized treatments other than R-CHOP, we were unable to perform subgroup analyses to compare the effect of upfront treatment (R-CHOP versus other therapy) on CR rate. Finally, our analysis included only de novo or newly diagnosed DLBCL. To date, only a few studies have reported on MYC and BCL2 co-expression in relapsed or refractory DLBCL [83,84], and therefore the prognostic value of double expressor status in this setting remains uncertain.

## 5. Conclusions

The pooled proportion of MYC/BCL2 double expressor status among patients with de novo DLBCL is 23% with an adjusted estimate of 31%. Patients with DLBCL without double expressor status had a 2.7 times higher probability of complete remission compared to patients with double expressor DLBCL. Double expressor status appears to be a valuable predictive biomarker in DLBCL.

## Figures and Tables

**Table 1:** Study characteristics.

First Author	Publication Year	Patient Enrollment Period	Institution	Country	Design	IHC Cut-Off Values for Protein Expression	
						MYC	BCL2
Abdulla M	2016	2002–2012	Uppsala University and University Hospital	Sweden	R	40%	70%
Barraclough A	2019	2002–2013	Various centers	Australia, United kingdom, Canada, Denmark	R	40%	50%
Birceanu Corobea A	2018	NA	Coltea Clinical Hospital	Romania	NA	40%	50%
Clark Schneider KM	2016	NA	Cleveland Clinic	USA	NA	40%	50%
Fogliatto L	2019	2011–2016	Hospital Santa Rita	Brazil	R	40%	40%
Friedberg JW	2014	2005–2010	Various centers	USA	Secondary analysis (Clinical trial, Phase 2)	40%	NA
Green TM	2012	2001–2008	Various centers	Denmark	R	40%	70%
Hori Y	2019	1999–2018	Kyushu University Hospital and its affiliated hospitals	Japan	R	40%	50%
Jesionek-Kupnicka D	2019	2017–2018	Medical University of Lodz	Poland	R	40%	50%
Johnson NA	2012	NA	Various centers	Various countries	NA	40%	50%
Jovanovic MP	2015	2001–2005	Clinical Center of Serbia	Serbia	R	30%	50%
Klanova M	2019	2011–2014	Various centers	Various countries	Secondary analysis (Clinical trial, Phase 3)	40%	50%
Li L	2018	2012–2015	Tianjin MedicalUniversity Cancer Institute and Hospital	China	R	40%	50%
Li M	2017	2004–2016	Various centers	China	NA	40%	50%
Liu Y	2017	2006–2016	Xi Jing Hospital and Tang Du Hospital in Xi’an	China	R	40%	50%
Lu TX	2015	2006–2014	First Affiliated Hospital of Nanjing Medical University	China	R	40%	50%
Ma Z	2019	2015–2017	First Affiliated Hospital of Xinjiang Medical University	China	R	50%	70%
Mohammed AA	2019	2011–2015	Zagazig University	Egypt	R	40%	50%
Molina TJ	2014	2003–2008	Various centers	France, Belgium, and Switzerland	Secondary analysis (Clinical trial, Phase 3)	40%	70%
Na HY	2019	1996–2016	Seoul National University Hospital, Seoul National University Bundang Hospital and Seoul National University Boramae Hospital	Korea	R	40%	50%
Pedersen MO	2017	2004–2008	NA	Denmark	R	40%	70%
Peroja P	2018	2003–2011	Oulu and Kuopio University Hospitals and Central Hospital of Central Finland	Finland	R	40%	70%
Perry AM	2014	NA	University ofNebraska Medical Center	USA	NA	50%	30%
Petrella T	2017	NA	Various centers	France, Belgium, Switzerland, and Portugal	Secondary analysis (Clinical trial, Phase 3)	40%	70%
Phang KC	2019	2004–2010	UKM Medical Centre	Malaysia	R	40%	70%
Rajnai H	2014	NA	Semmelweis University and the Leiden University Medical Center	Hungary, Netherlands	NA	30%	30%
Scott DW	2015	NA	British Columbia Cancer Agency	Canada	NA	40%	50%
Sha C	2019	NA	NA	Swiss and England	Secondary analysis (Clinical trial, Phase 3)	40%	50%
Staiger AM	2017	NA	Various centers	Germany and Switzerland	Secondary analysis (Clinical trial, Phase 3)	40%	50%
Suresh B	2019	2016–2017	Kidwai Cancer Institute	India	P	NA	NA
Takahashi H	2016	2001–2013	Nihon University School of Medicine	Japan	R	40%	50%
Teoh CS	2018	2012–2015	Hospital Pulau Pinang	Malaysia	R	40%	30%
Tessier-Cloutier B	2019	NA	NA	Sweden, Canada, USA	NA	40%	50%
Ting CY	2019	2012–2013	Hospital Ampang, Queen Elizabeth Hospital, Hospital Pulau Pinang and Sarawak General Hospital	Malaysia	R	40%	50%
Wang XJ	2017	2010–2015	Vanderbilt and MD Anderson Medical Center	USA	R	40%	50%
Xia B	2015	2005–2010	Tianjin Medical University Cancer Institute and Hospital	China	NA	30%	30%
Xie Y	2014	2002–2012	Los Angeles County and University of Southern California Medical Center	USA	R	40%	70%
Xu PP	2017	2002–2012	Shanghai Rui Jin Hospital	China	R	40%	70%
Yan LX	2014	2000–2012	Guangdong General Hospital	China	NA	40%	70%
Ye Q	2016	1998–2010	Various centers	Various countries	NA	70%	70%
Zhang Y	2018	2015–2016	Weifang People’s Hospital	China	R	40%	50%

R = retrospective; P = prospective; IHC = immunohistochemistry; NA = not available.

**Table 2:** Patient characteristics.

First Author	Patients (*N*)	Age (Range)	Male to Female Ratio	Clinical Setting
Abdulla M	188	64 (26–85)	1.4:1	De novo DLBCL
Barraclough A	175	62 (19–89)	1:1	PET-CT defined stage I/II DLBCL
Birceanu Corobea A	80	57.26 (19–87)	1.1:1	DLBCL
Clark Schneider KM	69	62 **	1:1	De novo DLBCL
Fogliatto L	83	64 (15–92)	1:1.4	DLBCL
Friedberg JW	84	64 (29–85)	1:1.2	Newly diagnosed advanced stage DLBCL
Green TM	193	64 (16–91)	1.4:1	De novo DLBCL
Hori Y	23	65 (38–84)	1.3:1	Primary colorectal DLBCL
Jesionek-Kupnicka D	217	68.73	1:1.2	DLBCL
Johnson NA	167 ^a^	62 (17–92)	NA	De novo DLBCL
Jovanovic MP	103	56 (17–87)	1:1.1	De novo DLBCL
Klanova M	688 ^b^	NA	NA	DLBCL in the phase 3 GOYA study
Li L	212	58.5 (21–86)	1.2:1	Newly diagnosed DLBCL
Li M	35	62 (23–89)	2:1	Anaplastic DLBCL
Liu Y	100	NA	1.1:1	Primary gastrointestinal DLBCL
Lu TX	246	NA	NA	De novo DLBCL
Ma Z	98	55 (8–76)	1.3:1	De novo DLBCL
Mohammed AA	90	58 (25–90)	1.2:1	De novo DLBCL
Molina TJ	379	NA	NA	De novo DLBCL
Na HY	195	NA	1.3:1	De novo DLBCL
Pedersen MO	103	NA (18–60)	1.3:1	De novo high-risk DLBCL
Peroja P	155	NA	1.2:1	De novo DLBCL
Perry AM	106 ^c^	61 (19–89)	1.2:1	De novo DLBCL
Petrella T	285	70 (59–80)	2.2:1	Untreated elderly patients with DLBCL
Phang KC	141	NA	NA	DLBCL
Rajnai H	41	50 (11–78)	2.4:1	Primary bone DLBCL
Scott DW	344	64 (16–92)	1.6:1	De novo DLBCL
Sha C	355 ^d^	NA	NA	Newly diagnosed DLBCL
Staiger AM	414	NA	1:1 (RICOVER-60 Trial), 1.4:1 (R-MegaCHOEP Trial)	Untreated DLBCL
Suresh B	21	46 (27–69)	2.5:1	Primary gastrointestinal DLBCL
Takahashi H	40	53 (19–68)	1:1.3	De novo DLBCL with high/high-intermediate risk by aaIPI
Teoh CS	104	NA	1:1	DLBCL
Tessier-Cloutier B	20	58 (48–66)	9:1	SLE diagnosed with DLBCL
Ting CY	120	54.1 (14.6) **	1.1:1	De novo DLBCL
Wang XJ	201	64 (18–92)	1.9:1	De novo DLBCL
Xia B	60	57 (23–79)	1.1:1	Primary gastrointestinal DLBCL
Xie Y	85	54 (20–89)	1.5:1	De novo DLBCL
Xu PP	470 ^e^	NA	NA	De novo DLBCL
Yan LX	336	57 (7–87)	1.4:1	De novo DLBCL
Ye Q	898	64 (16–95)	NA	De novo DLBCL
Zhang Y	42	58.9 (43–80)	1.6:1	Newly diagnosed DLBCL

^a^ Training cohort in the study; ^b^ Among 1418 patients, MYC/BCL2 protein expression was available from 688 patients; ^c^ Training cohort in the study; ^d^ Among 928 patients, a subset of 355 patients was investigated for MYC/BCL2 protein expression; ^e^ Among 680 patients, MYC/BCL2 protein expression was available from 470 patients; ** Age is presented as mean (±standard deviation). Other age data are presented as median. NA = not available.

**Table 3:** Summary of the meta-analytic pooled prevalence for various outcomes among the included studies.

Outcome	No. of Studies	Summary Estimate			*p* Value for Publication Bias ^c^	Trim-and-Fill Estimate	
		Pooled Proportion (%) (95% CI)	*p* Value for Hetero-Geneity ^a^	I^2^ (%) ^b^		No. of Missing Studies	Adjusted Pooled Proportion (95% CI)
Double expressor (MYC+, BCL2+)	41	23 (20–26)	<0.001	90	<0.001	16	31 (27–36)
MYC protein expression	29	34 (30–39)	<0.001	90	0.421		
BCL2 protein expression	30	58 (53–62)	<0.001	90	0.585		
Double expressor (MYC > 40%, BCL2 > 50%)	21	20 (16–26)	<0.001	92	<0.001	9	32 (26–39)
Double expressor (MYC > 40%, BCL2 > 70%)	10	27 (23–32)	<0.001	79	0.07	3	30 (25–35)
Double expressor in Asian countries	16	23 (17–28)	<0.001	87	0.05	5	30 (23–37)
Double expressor in Europe	12	21 (17–27)	<0.001	87	0.006	5	29 (23–37)
Double expressor in North America	6	29 (20–39)	<0.001	88	NA ^d^		
Double expressor in studies with median age ≥ 60.3	16	25 (19–31)	<0.001	93	0.16		
Double expressor in studies with median age < 60.3	14	20 (16–24)	<0.001	63	0.03	6	25 (21–30)

^a^*p* value was determined by the Q test with *p* < 0.05 representing substantial heterogeneity. ^b^ Inconsistency index >50% indicates substantial heterogeneity. ^c^ *p* values were calculated by Egger’s test with <0.1 representing significant publication bias. ^d^ A *p* value is not available due to the small number of included studies (*n* < 10).

## Data Availability

The databases for the analyses of this study are available on request from the corresponding author.

## Supplementary Materials

The following are available online at https://www.mdpi.com/article/10.3390/cancers13133369/s1, Figure S1: Funnel plots for pooled proportion of double protein expression of MYC and BCL2, Table S1: Newcastle-Ottawa Quality Assessment Scale of included studies in the synthesis of double expressor status, Table S2: Patient characteristics of included studies in the synthesis of double expressor status, Table S3: IHC protocols, Table S4: Study and patient characteristics from articles reporting DHITsig status in DLBCL.
