# Supplementary material for: The Incidence and Treatment Response of Double Expression of MYC and BCL2 in Patients with Diffuse Large B-Cell Lymphoma: A Systematic Review and Meta-Analysis
Source: Cancers (Basel). 2021 Jul 5;13(13):3369. doi: 10.3390/cancers13133369 (PMC8268769; doi:10.3390/cancers13133369)
Supplement: Supplementary file 1 [file cancers-13-03369-s001.zip › cancers-1198823-supplementary.pdf]

Supplementary Materials: The Incidence and Treatment Response of Double Expression of MYC and BCL2 in Patients with Diffuse Large B-Cell Lymphoma: A Systematic Review and Meta-Analysis

Jisun Hwang, Chong Hyun Suh, Kyung Won Kim, Ho Sung Kim, Austin I. Kim, Jeffrey W. Craig, Ke Xun Chen, Joel Roberson, Jeffrey P. Guenette and Raymond Y. Huang

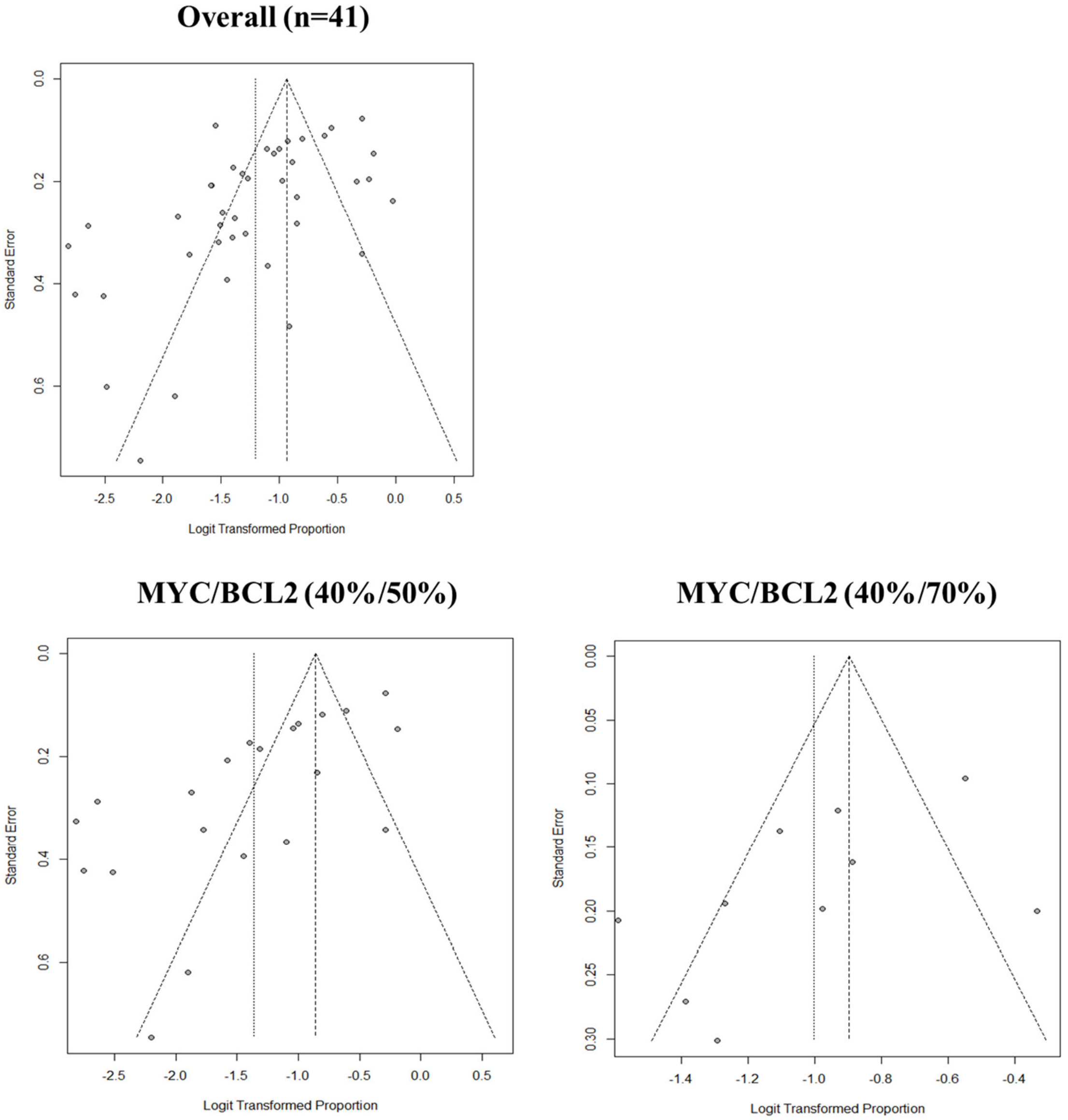

Figure S1. Funnel plots for pooled proportion of double protein expression of MYC and BCL2. Funnel plot and Egger's test revealed substantial publication bias in overall pooled proportion ($p < 0.001$) and subgroups using ≥40%/50% cutoffs ($p < 0.001$) and ≥40%/70% cutoffs ($p = 0.07$).

Table S1. Newcastle-Ottawa Quality Assessment Scale of included studies in the synthesis of double expressor status.

Reference	Design	Selection	Comparability	Outcome	Total Score
Abdulla et al. (2016)[1]	Cohort	4	0	2	6
Barraclough et al. (2019)[2]	Cohort	4	2	3	9
Birceanu Corobea et al. (2018)[3]	Cohort	4	0	3	7
Clark Schneider et al. (2016)[4]	Cohort	4	2	2	8
Fogliatto et al. (2019)[5]	Cohort	4	2	2	8
Friedberg et al. (2014)[6]	Secondary analysis	4	0	2	6
Green et al. (2012)[7]	Cohort	4	2	3	9
Hori et al. (2019)[8]	Cohort	4	0	3	7
Jesionek-Kupnicka et al. (2019)[9]	Cohort	4	2	1	7
Johnson et al. (2012)[10]	Cohort	4	2	2	8
Jovanovic et al. (2015)[11]	Cohort	4	0	2	6
Klanova et al. (2019)[12]	Secondary analysis	4	2	2	8
Li et al. (2018)[13]	Cohort	4	0	2	6
Li et al. (2017)[14]	Cohort	4	2	2	8
Liu et al. (2017)[15]	Cohort	4	2	2	8
Lu et al. (2015)[16]	Cohort	4	2	3	9
Ma et al. (2019)[17]	Cohort	4	2	2	8
Mohammed et al. (2019)[18]	Cohort	4	2	3	9
Molina et al. (2014)[19]	Secondary analysis	4	2	3	9
Na et al. (2019)[20]	Cohort	4	2	2	8
Pedersen et al. (2017)[21]	Cohort	4	2	2	8
Peroja et al. (2018)[22]	Cohort	4	2	2	8
Perry et al. (2014)[23]	Cohort	4	0	3	7
Petrella et al. (2017)[24]	Secondary analysis	4	2	2	8
Phang et al. (2019)[25]	Cohort	4	2	2	8
Rajnai et al. (2014)[26]	Cohort	4	2	3	9
Scott et al. (2015)[27]	Cohort	4	2	2	8
Sha et al. (2019)[28]	Secondary analysis	4	0	2	6
Staiger et al. (2017)[29]	Secondary analysis	4	2	2	8
Suresh et al. (2019)[30]	Cohort	4	0	2	6
Takahashi et al. (2016)[31]	Cohort	4	2	3	9
Teoh et al. (2018)[32]	Cohort	4	2	3	9
Tessier-Cloutier et al. (2019)[33]	Cohort	4	0	2	6
Ting et al. (2019)[34]	Cohort	4	2	2	8
Wang et al. (2017) *[35]	Cohort	4	2	3	9
Xia et al. (2015)[36]	Cohort	4	2	3	9
Xie et al. (2014)[37]	Cohort	4	0	3	7
Xu et al. (2017)[38]	Cohort	4	2	3	9
Yan et al. (2014)[39]	Cohort	4	2	2	8
Ye et al. (2016)[40]	Cohort	4	2	2	8
Zhang Y et al. (2018)[41]	Cohort	4	2	1	7
Wang et al. (2015)[42]	Cohort	3	0	2	5

Note–overall quality of a study: 8–9, very good; 6–7, good; 4–5, satisfactory; 0–3, unsatisfactory. * A study by Wang et al. selected patients whose outcome were already present at start of study. The study received five scores ("satisfactory" rating) and was excluded in the systematic review and meta-analyses.

Table S2. Patient characteristics of included studies in the synthesis of double expressor status.

First Author	IPI Category	Ann Arbor Stage	Elevated LDH	Treatment Arm
Abdulla M	† 0 (19%), 1(24%), 2 (38%), 3(8%)	1(21%), 2(15%), 3(18%), 4(37%)	61%	R-CHOP
Barraclough A	NA	1(57%), 2(43%)	31%	R-CHOP or R-CHOP-like regimens
Birceanu Corobea A	NA	1–2(37.5%), 3–4(62.5%)	NA	R-CHOP
Clark Schneider KM	0–2 (55%), 3–5 (35%)	1–2(45%), 3–4(51%)	NA	R-CHOP
Fogliatto L	‡ 0 (10%), 1–2 (61.4%), 3–5 (28.6%)	NA	50%	R-CHOP or R-CHOP-like regimens
Friedberg JW	0–1 (24%), 2 (32%), 3 (32%), 4–5 (12%)	2–4(100%)	NA	R-CHOP with Iodine-131 Tositumomab consolidation (radioimmunotherapy)
Green TM	3–5 (34%)	3–4(48%)	48%	R-CHOP
Hori Y	0–1 (48%), 2 (22%), 3 (22%), 4–5 (7%)	* I-II1 (48%), II2, IIE, and IV (52%)		CHOP-based regimen with or without rituximab
Jesion-ek-Kupnicka D	NA	NA	NA	NA
Johnson NA	0–1 (47%), 2 (27%), 3 (14%), 4–5 (12%)	>2 (50%)	40%	R-CHOP
Jovanovic MP	0–2 (51%), 3–5 (49%)	1–2 (29%), 3–4 (71%)	66%	R-CHOP or CHOP protocols
Klanova M	NA	NA	NA	G-CHOP or R-CHOP
Li L	0–2 (71.7%), 3–5 (26.4%)	1–2 (46.7%), 3–4 (47.2%)	43.90%	R-CHOP-like regimens
Li M	≥3 (65%)	3–4 (85%)	62%	CHOP or EPOCH plus rituximab or not
Liu Y	0–1 (50%), 2–5 (50%)	NA	NA	NA
Lu TX	NA	NA	NA	R-CHOP like regimens in 141 patients, NA in others
Ma Z	0–2 (54.08%), 3–5 (45.92%)	1–2 (31.63), 3–4 (68.37)	44.90%	R-CHOP or R-CHOP-like regimens
Mohammed AA	0–1(15.6%), 2(25.6%), 3(36.7%), 4–5(22.2%)	1(15.6%), 2(27.8%), 3(38.9%), 4(17.8%)	NA	R-CHOP
Molina TJ	NA	NA	NA	R-ACVBP vs. R-CHOP (LNH 03-2B trial)
Na HY	0–2 (63.5%), 3–5 (36.4%)	1–2 (54.8%), 3–4 (45.1%)	50.20%	R-CHOP, CHOP, other chemotherapy
Pedersen MO	†2 (78%), 3 (22%)	1 (1%), 2(5%), 3(42%), 4(50%)	96%	R-CHOP or R-CHOEP
Peroja P	0–1(38%), 2–3 (48%), 4–5 (11%)	3–4 (53%)	57%	R-CHOP like regimens
Perry AM	0–2(75%), 3–5(25%)	1–2(53%), 3–4(48%)	47%	Rituximab and CHOP or CHOP-like regimens
Petrella T	0(0%), 1(1%), 2(23%), 3(38%), 4(28%), 5(10%)	3–4(90%)		R-CHOP (LNH03-6B trial)
Phang KC	NA	NA	NA	NA
Rajnai H	0–2(58.5%), 3–5(17%)	1(68.2%), 2(9.7%), 4(21.9%)	NA	R-CHOP,CHOP,CHOP-like,unknown
Scott DW	0–1(35%), 2–3(46%), 4–5(19%)	1–2(49%), 3–4(51%)	52%	R-CHOP
Sha C	NA	NA	NA	R-CHOP and RB-CHOP (REMoDL-B trial)

Staiger AM	NA	NA	NA	CHOP-14 with or without rituximab (RICOVER-60 trial), CHOEP-14 or Mega-CHOEP-21 (R-MegaCHOEP trial)
Suresh B	NA	* IE (9.5%), IIE (66.7%),IV(23.8%)	NA	CHOP based chemotherapy +/− rituximab
Takahashi H	†2(47.5%), 3(52.5%)	1–2(0%), 3–4(100%)	97.50%	R-Double-CHOP regimen
Teoh CS	NA	NA	NA	R-CHOP
Tessier-Cloutier B	NA	NA	NA	NA
Ting CY	>2 (41.7%)	3–4(56.7%)	74.20%	Various (R-CHOP like, CHOP-like, MTX-based regimen, EPOCH)
Wang XJ	≥3 (56.3%)	3–4(65.9%)	57%	R-CHOP, R-EPOCH, R-hyper-CVAD/Ara-C/MTX
Xia B	0–2(83.3%), 3–5(16.6%)	*I-II2 (60%), IIE-IV (40%)	43.30%	R-CHOP-like regimen
Xie Y	0(20%), 1(26%),2(19%),3(13%),4(19%),5(4%)	1(21%), 2(26%), 3(12%), 4(41%)	53%	R-CHOP, R-CHOP-like regimen
Xu PP	NA	NA	NA	R-CHOP
Yan LX	0–1(51%), 2(25%), 3(18%), 4–5(7%)	3–4(46%)	38%	R-CHOP or CHOP
Ye Q	NA	NA	NA	R-CHOP
Zhang Y	NA	NA	NA	NA

† Age-adjusted IPI; ‡ Revised IPI; * Lugano staging system.

Table S3. IHC protocols.

Reference	Details of IHC Protocol			Reviewer of IHC Stains
	Platform	Antibody	Staining Conditions and Other Details	
Abdulla et al. (2016)[1]	1	1	0	P
Barraclough et al. (2019)[2]	0	0	0	0
Birceanu Corobea et al. (2018)[3]	0	1	1	P
Clark Schneider et al. (2016)[4]	1	1	1	P,HP
Fogliatto et al. (2019)[5]	0	1	0	P
Friedberg et al. (2014)[6]	0	1	0	0
Green et al. (2012)[7]	1	1	0	P
Hori et al. (2019)[8]	0	1	1	P
Jesionek-Kupnicka et al. (2019)[9]	1	1	0	0
Johnson et al. (2012)[10]	1	1	0	P
Jovanovic et al. (2015)[11]	0	1	0	HP
Klanova et al. (2019)[12]	1	1	0	0
Li et al. (2018)[13]	0	1	0	P
Li et al. (2017)[14]	1	1	1	P
Liu et al. (2017)[15]	1	1	0	P
Lu et al. (2015)[16]	0	1	0	0
Ma et al. (2019)[17]	0	1	1	HP
Mohammed et al. (2019)[18]	1	1	1	0
Molina et al. (2014)[19]	1	1	1	P
Na et al. (2019)[20]	1	1	1	HP
Pedersen et al. (2017)[21]	1	1	1	HP
Peroja et al. (2018)[22]	0	1	1	HP
Perry et al. (2014)[23]	1	1	1	HP
Petrella et al. (2017)[24]	1	1	1	0
Phang et al. (2019)[25]	0	1	1	HP
Rajnai et al. (2014)[26]	0	1	0	HP
Scott et al. (2015)[27]	1	1	1	HP
Sha et al. (2019)[28]	0	1	0	P
Staiger et al. (2017)[29]	0	1	1	HP
Suresh et al. (2019)[30]	0	1	0	0
Takahashi et al. (2016)[31]	0	1	0	HP
Teoh et al. (2018)[32]	0	1	0	P
Tessier-Cloutier et al. (2019)[33]	0	1	0	P
Ting et al. (2019)[34]	1	1	1	P
Wang et al. (2017)[35]	0	1	0	0
Xia et al. (2015)[36]	0	1	1	HP
Xie et al. (2014)[37]	1	1	1	P
Xu et al. (2017)[38]	0	1	1	0
Yan et al. (2014)[39]	1	1	1	P
Ye et al. (2016)[40]	0	1	0	0
Zhang Y et al. (2018)[41]	1	1	0	P

1 = Yes; 0 = not available; P = pathologist(s); HP = hematopathologist(s).

Table S4. Study and patient characteristics from articles reporting DHITsig status in DLBCL.

First Author	Publication Year	Patient Enrollment Period	Institution	Country	Design	Patients (N)	Age (Range)	Male to Female Ratio	Clinical Setting
Nguyen et al.[43]	2021	2000–2016	City of Hope National Medical Center and from the province of Manitoba/ CancerCare Manitoba	USA	NA	247	60	1.4:1	de novo DLBCL
Isaksen et al.[44]	2021	2000–2012	23 North American academic medical centers	North America	Secondary analysis (Clinical trial, Phase 2)	90	55 (22–65)	66:34	high-risk de novo DLBCL
Ennishi et al.[45]	2018	1985–2011	BC Cancer	Canada	NA	157	62 (35–79) in DHITsig-positive, 62 (19–92) in DHITsig-negative group	95:62	de novo GCB-DLBCL

References

1. Abdulla, M.; Laszlo, S.; Triumf, J.; Hedstrom, G.; Berglund, M.; Enblad, G.; Amini, R.M. A population-based study of cellular markers in R-CHOP treated diffuse large B-cell lymphoma patients. *Acta oncologica (Stockholm, Sweden)* **2016**, *55*, 1126-1131, doi:10.1080/0284186x.2016.1189093.
2. Barraclough, A.; Alzahrani, M.; Ettrup, M.S.; Bishton, M.; van Vliet, C.; Farinha, P.; Gould, C.; Birch, S.; Sehn, L.H.; Sovani, V., et al. COO and MYC/BCL2 status do not predict outcome among patients with stage I/II DLBCL: a retrospective multicenter study. *Blood advances* **2019**, *3*, 2013-2021, doi:10.1182/bloodadvances.2019000251.
3. Birceanu Corobea A, E.A., Rosianu C, Gheorghe M, Birceanu G, Copca N, Sajin M, Lupu A. The survival impact of combined expression of MYC, BCL2 and BCL6 in diffuse large B cell lymphoma patients. *Arch Balk Med Union* **2018**, *53*, 232-238, doi:10.31688/ABMU.2018.53.2.09.
4. Clark Schneider, K.M.; Banks, P.M.; Collie, A.M.; Lanigan, C.P.; Manilich, E.; Durkin, L.M.; Hill, B.T.; Hsi, E.D. Dual expression of MYC and BCL2 proteins predicts worse outcomes in diffuse large B-cell lymphoma. *Leukemia & lymphoma* **2016**, *57*, 1640-1648, doi:10.3109/10428194.2015.1101099.
5. Fogliatto, L.; Grokoski, K.C.; Strey, Y.M.; Vanelli, T.; Fraga, C.; Barra, M.B.; Pinto, F.C.; Bendit, I.; Bica, C.G. Prognostic impact of MYD88 mutation, proliferative index and cell origin in diffuse large B cell lymphoma. *Hematology, transfusion and cell therapy* **2019**, *41*, 50-56, doi:10.1016/j.htct.2018.05.014.
6. Friedberg, J.W.; Unger, J.M.; Burack, W.R.; Gopal, A.K.; Raju, R.N.; Nademanee, A.P.; Kaminski, M.S.; Li, H.; Press, O.W.; Miller, T.P., et al. R-CHOP with iodine-131 tositumomab consolidation for advanced stage diffuse large B-cell lymphoma (DLBCL): SWOG S0433. *British journal of haematology* **2014**, *166*, 382-389, doi:10.1111/bjh.12906.
7. Green, T.M.; Young, K.H.; Visco, C.; Xu-Monette, Z.Y.; Orazi, A.; Go, R.S.; Nielsen, O.; Gadeberg, O.V.; Mourits-Andersen, T.; Frederiksen, M., et al. Immunohistochemical double-hit score is a strong predictor of outcome in patients with diffuse large B-cell lymphoma treated with rituximab plus cyclophosphamide, doxorubicin, vincristine, and prednisone. *Journal of clinical oncology : official journal of the American Society of Clinical Oncology* **2012**, *30*, 3460-3467, doi:10.1200/jco.2011.41.4342.
8. Hori, Y.; Yamamoto, H.; Nozaki, Y.; Torisu, T.; Fujiwara, M.; Taguchi, K.; Nishiyama, K.; Nakamura, S.; Kitazono, T.; Oda, Y. Colorectal diffuse large B-cell lymphoma: molecular subclassification and prognostic significance of immunoglobulin gene translocation. *Human pathology* **2019**, 10.1016/j.humpath.2019.09.003, doi:10.1016/j.humpath.2019.09.003.
9. Jesionek-Kupnicka, D.; Braun, M.; Robak, T.; Kuncman, W.; Kordek, R. A large single-institution retrospective analysis of aggressive B-cell lymphomas according to the 2016/2017 WHO classification. *Advances in clinical and experimental medicine : official organ Wroclaw Medical University* **2019**, *28*, 1359-1365, doi:10.17219/acem/109200.
10. Johnson, N.A.; Slack, G.W.; Savage, K.J.; Connors, J.M.; Ben-Neriah, S.; Rogic, S.; Scott, D.W.; Tan, K.L.; Steidl, C.; Sehn, L.H., et al. Concurrent expression of MYC and BCL2 in diffuse large B-cell lymphoma treated with rituximab plus cyclophosphamide, doxorubicin, vincristine, and prednisone. *Journal of clinical oncology : official journal of the American Society of Clinical Oncology* **2012**, *30*, 3452-3459, doi:10.1200/jco.2011.41.0985.
11. Jovanovic, M.P.; Mihaljevic, B.; Jakovic, L.; Martinovic, V.C.; Fekete, M.D.; Andjelic, B.; Antic, D.; Bogdanovic, A.; Boricic, N.; Terzic, T., et al. BCL2 positive and BCL6 negative diffuse large B cell lymphoma patients benefit from R-CHOP therapy irrespective of germinal and non germinal center B cell like subtypes. *Journal of B.U.ON. : official journal of the Balkan Union of Oncology* **2015**, *20*, 820-828.
12. Klanova, M.; Sehn, L.H.; Bence-Bruckler, I.; Cavallo, F.; Jin, J.; Martelli, M.; Stewart, D.; Vitolo, U.; Zaja, F.;

Zhang, Q., et al. Integration of cell of origin into the clinical CNS International Prognostic Index improves CNS relapse prediction in DLBCL. *Blood* **2019**, *133*, 919-926, doi:10.1182/blood-2018-07-862862.

13. Li, L.; Zhang, X.; Zhang, T.; Song, Z.; Hu, G.; Li, W.; Li, L.; Qiu, L.; Qian, Z.; Zhou, S., et al. Prognostic Significance of BCL-2 and BCL-6 Expression in MYC-positive DLBCL. *Clinical lymphoma, myeloma & leukemia* **2018**, *18*, e381-e389, doi:10.1016/j.clml.2018.06.010.
14. Li, M.; Liu, Y.; Wang, Y.; Chen, G.; Chen, Q.; Xiao, H.; Liu, F.; Qi, C.; Yu, Z.; Li, X., et al. Anaplastic Variant of Diffuse Large B-cell Lymphoma Displays Intricate Genetic Alterations and Distinct Biological Features. *The American journal of surgical pathology* **2017**, *41*, 1322-1332, doi:10.1097/pas.0000000000000836.
15. Liu, Y.; Yu, K.; Li, M.; Zeng, K.; Wei, J.; Li, X.; Liu, Y.; Zhao, D.; Fan, L.; Yu, Z., et al. EZH2 overexpression in primary gastrointestinal diffuse large B-cell lymphoma and its association with the clinicopathological features. *Human pathology* **2017**, *64*, 213-221, doi:10.1016/j.humpath.2017.04.011.
16. Lu, T.X.; Fan, L.; Wang, L.; Wu, J.Z.; Miao, K.R.; Liang, J.H.; Gong, Q.X.; Wang, Z.; Young, K.H.; Xu, W., et al. MYC or BCL2 copy number aberration is a strong predictor of outcome in patients with diffuse large B-cell lymphoma. *Oncotarget* **2015**, *6*, 18374-18388, doi:10.18632/oncotarget.4073.
17. Ma, Z.; Niu, J.; Cao, Y.; Pang, X.; Cui, W.; Zhang, W.; Li, X. Clinical significance of 'double-hit' and 'double-expression' lymphomas. *Journal of clinical pathology* **2020**, *73*, 126-138, doi:10.1136/jclinpath-2019-206199.
18. Mohammed, A.A.; Rashed, H.E.; Abdelrahman, A.E.; Obaya, A.A.; Toam, M.; Abdel Nour, H.M.; Abdelhamid, M.I.; Elsayed, F.M. C-MYC and BCL2: Correlation between Protein Over-Expression and Gene Translocation and Impact on Outcome in Diffuse Large B Cell Lymphoma. *Asian Pacific journal of cancer prevention : APJCP* **2019**, *20*, 1463-1470, doi:10.31557/apjcp.2019.20.5.1463.
19. Molina, T.J.; Canioni, D.; Copie-Bergman, C.; Recher, C.; Briere, J.; Haioun, C.; Berger, F.; Ferme, C.; Copin, M.C.; Casasnovas, O., et al. Young patients with non-germinal center B-cell-like diffuse large B-cell lymphoma benefit from intensified chemotherapy with ACVBP plus rituximab compared with CHOP plus rituximab: analysis of data from the Groupe d'Etudes des Lymphomes de l'Adulte/lymphoma study association phase III trial LNH 03-2B. *Journal of clinical oncology : official journal of the American Society of Clinical Oncology* **2014**, *32*, 3996-4003, doi:10.1200/jco.2013.54.9493.
20. Na, H.Y.; Choe, J.Y.; Shin, S.A.; Kim, H.J.; Han, J.H.; Kim, H.K.; Oh, S.H.; Kim, J.E. Characteristics of CD5-positive diffuse large B-cell lymphoma among Koreans: High incidence of BCL2 and MYC double-expressors. *PloS one* **2019**, *14*, e0224247, doi:10.1371/journal.pone.0224247.
21. Pedersen, M.O.; Gang, A.O.; Brown, P.; Pedersen, M.; Knudsen, H.; Nielsen, S.L.; Poulsen, T.; Wirenfeldt Klausen, T.; Hogdall, E.; Norgaard, P. Real world data on young patients with high-risk diffuse large B-cell lymphoma treated with R-CHOP or R-CHOEP - MYC, BCL2 and BCL6 as prognostic biomarkers. *PloS one* **2017**, *12*, e0186983, doi:10.1371/journal.pone.0186983.
22. Peroja, P.; Pedersen, M.; Mantere, T.; Norgaard, P.; Peltonen, J.; Haapasaari, K.M.; Bohm, J.; Jantunen, E.; Turpeenniemi-Hujanen, T.; Rapakko, K., et al. Mutation of TP53, translocation analysis and immunohistochemical expression of MYC, BCL-2 and BCL-6 in patients with DLBCL treated with R-CHOP. *Scientific reports* **2018**, *8*, 14814, doi:10.1038/s41598-018-33230-3.
23. Perry, A.M.; Alvarado-Bernal, Y.; Laurini, J.A.; Smith, L.M.; Slack, G.W.; Tan, K.L.; Sehn, L.H.; Fu, K.; Aoun, P.; Greiner, T.C., et al. MYC and BCL2 protein expression predicts survival in patients with diffuse large B-cell lymphoma treated with rituximab. *British journal of haematology* **2014**, *165*, 382-391, doi:10.1111/bjh.12763.
24. Petrella, T.; Copie-Bergman, C.; Briere, J.; Delarue, R.; Jardin, F.; Ruminy, P.; Thieblemont, C.; Figeac, M.; Canioni, D.; Feugier, P., et al. BCL2 expression but not MYC and BCL2 coexpression predicts survival in elderly patients with diffuse large B-cell lymphoma independently of cell of origin in the phase 3 LNH03-6B

trial. *Annals of oncology : official journal of the European Society for Medical Oncology* **2017**, *28*, 1042-1049, doi:10.1093/annonc/mdx022.
25. Phang, K.C.; Hussin, N.H.; Abdul Rahman, F.; Tizen, N.M.S.; Mansoor, A.; Masir, N. Characterisation of immunogenotypes of diffuse large B-cell lymphoma. *The Malaysian journal of pathology* **2019**, *41*, 101-124.
26. Rajnai, H.; Heyning, F.H.; Koens, L.; Sebestyen, A.; Andrikovics, H.; Hogendoorn, P.C.; Matolcsy, A.; Szepesi, A. The density of CD8+ T-cell infiltration and expression of BCL2 predicts outcome of primary diffuse large B-cell lymphoma of bone. *Virchows Archiv : an international journal of pathology* **2014**, *464*, 229-239, doi:10.1007/s00428-013-1519-9.
27. Scott, D.W.; Mottok, A.; Ennishi, D.; Wright, G.W.; Farinha, P.; Ben-Neriah, S.; Kridel, R.; Barry, G.S.; Hother, C.; Abrisqueta, P., et al. Prognostic Significance of Diffuse Large B-Cell Lymphoma Cell of Origin Determined by Digital Gene Expression in Formalin-Fixed Paraffin-Embedded Tissue Biopsies. *Journal of clinical oncology : official journal of the American Society of Clinical Oncology* **2015**, *33*, 2848-2856, doi:10.1200/jco.2014.60.2383.
28. Sha, C.; Barrans, S.; Cucco, F.; Bentley, M.A.; Care, M.A.; Cummin, T.; Kennedy, H.; Thompson, J.S.; Uddin, R.; Worrillow, L., et al. Molecular High-Grade B-Cell Lymphoma: Defining a Poor-Risk Group That Requires Different Approaches to Therapy. *Journal of clinical oncology : official journal of the American Society of Clinical Oncology* **2019**, *37*, 202-212, doi:10.1200/jco.18.01314.
29. Staiger, A.M.; Ziepert, M.; Horn, H.; Scott, D.W.; Barth, T.F.E.; Bernd, H.W.; Feller, A.C.; Klapper, W.; Szczepanowski, M.; Hummel, M., et al. Clinical Impact of the Cell-of-Origin Classification and the MYC/ BCL2 Dual Expresser Status in Diffuse Large B-Cell Lymphoma Treated Within Prospective Clinical Trials of the German High-Grade Non-Hodgkin's Lymphoma Study Group. *Journal of clinical oncology : official journal of the American Society of Clinical Oncology* **2017**, *35*, 2515-2526, doi:10.1200/jco.2016.70.3660.
30. Suresh, B.; Asati, V.; Lakshmaiah, K.C.; Babu, G.; Lokanatha, D.; Jacob, L.A.; Lokesh, K.N.; Rudresh, A.H.; Rajeev, L.K.; Smitha, S., et al. Primary gastrointestinal diffuse large B-cell lymphoma: A prospective study from South India. *South Asian journal of cancer* **2019**, *8*, 57-59, doi:10.4103/sajc.sajc_52_18.
31. Takahashi, H.; Miura, K.; Nakagawa, M.; Sugitani, M.; Amano, Y.; Kurita, D.; Sakagami, M.; Ohtake, S.; Uchino, Y.; Kodaira, H., et al. Negative impact of concurrent overexpression of MYC and BCL2 in patients with advanced diffuse large B-cell lymphoma treated with dose-intensified immunochemotherapy. *Leukemia & lymphoma* **2016**, *57*, 2784-2790, doi:10.3109/10428194.2016.1167205.
32. Teoh, C.S.; Lee, S.Y.; Chiang, S.K.; Chew, T.K.; Goh, A.S. Impact of Double Expression of C-MYC/BCL2 Protein and Cell of Origin Subtypes on the Outcome among Patients with Diffuse Large B-Cell Lymphoma: a Single Asian Center Experience. *Asian Pacific journal of cancer prevention : APJCP* **2018**, *19*, 1229-1236, doi:10.22034/apjcp.2018.19.5.1229.
33. Tessier-Cloutier, B.; Twa, D.D.; Baecklund, E.; Gascoyne, R.; Johnson, N.A.; Backlin, C.; Kamen, D.L.; Clarke, A.E.; Ramsey-Goldman, R.; Lee, J.L., et al. Cell of origin in diffuse large B-cell lymphoma in systemic lupus erythematosus: molecular and clinical factors associated with survival. *Lupus science & medicine* **2019**, *6*, e000324, doi:10.1136/lupus-2019-000324.
34. Ting, C.Y.; Chang, K.M.; Kuan, J.W.; Sathar, J.; Chew, L.P.; Wong, O.J.; Yusuf, Y.; Wong, L.; Samsudin, A.T.; Pana, M., et al. Clinical Significance of BCL2, C-MYC, and BCL6 Genetic Abnormalities, Epstein-Barr Virus Infection, CD5 Protein Expression, Germinal Center B Cell/Non-Germinal Center B-Cell Subtypes, Co-expression of MYC/BCL2 Proteins and Co-expression of MYC/BCL2/BCL6 Proteins in Diffuse Large B-Cell Lymphoma: A Clinical and Pathological Correlation Study of 120 Patients. *International journal of medical sciences* **2019**, *16*, 556-566, doi:10.7150/ijms.27610.
35. Wang, X.J.; Medeiros, L.J.; Bueso-Ramos, C.E.; Tang, G.; Wang, S.; Oki, Y.; Desai, P.; Khoury, J.D.; Miranda, R.N.; Tang, Z., et al. P53 expression correlates with poorer survival and augments the negative prognostic

effect of MYC rearrangement, expression or concurrent MYC/BCL2 expression in diffuse large B-cell lymphoma. *Modern pathology : an official journal of the United States and Canadian Academy of Pathology, Inc* **2017**, *30*, 194-203, doi:10.1038/modpathol.2016.178.

36. Xia, B.; Zhang, L.; Guo, S.Q.; Li, X.W.; Qu, F.L.; Zhao, H.F.; Zhang, L.Y.; Sun, B.C.; You, J.; Zhang, Y.Z. Coexpression of MYC and BCL-2 predicts prognosis in primary gastrointestinal diffuse large B-cell lymphoma. *World journal of gastroenterology* **2015**, *21*, 2433-2442, doi:10.3748/wjg.v21.i8.2433.
37. Xie, Y.; Bulbul, M.A.; Ji, L.; Inouye, C.M.; Groshen, S.G.; Tulpule, A.; O'Malley, D.P.; Wang, E.; Siddiqi, I.N. p53 expression is a strong marker of inferior survival in de novo diffuse large B-cell lymphoma and may have enhanced negative effect with MYC coexpression: a single institutional clinicopathologic study. *American journal of clinical pathology* **2014**, *141*, 593-604, doi:10.1309/ajcpphmz6vhf0wqv.
38. Xu, P.P.; Zhong, H.J.; Huang, Y.H.; Gao, X.D.; Zhao, X.; Shen, Y.; Cheng, S.; Huang, J.Y.; Chen, S.J.; Wang, L., et al. B-cell Function Gene Mutations in Diffuse Large B-cell Lymphoma: A Retrospective Cohort Study. *EBioMedicine* **2017**, *16*, 106-114, doi:10.1016/j.ebiom.2017.01.027.
39. Yan, L.X.; Liu, Y.H.; Luo, D.L.; Zhang, F.; Cheng, Y.; Luo, X.L.; Xu, J.; Cheng, J.; Zhuang, H.G. MYC expression in concert with BCL2 and BCL6 expression predicts outcome in Chinese patients with diffuse large B-cell lymphoma, not otherwise specified. *PloS one* **2014**, *9*, e104068, doi:10.1371/journal.pone.0104068.
40. Ye, Q.; Xu-Monette, Z.Y.; Tzankov, A.; Deng, L.; Wang, X.; Manyam, G.C.; Visco, C.; Montes-Moreno, S.; Zhang, L.; Dybkaer, K., et al. Prognostic impact of concurrent MYC and BCL6 rearrangements and expression in de novo diffuse large B-cell lymphoma. *Oncotarget* **2016**, *7*, 2401-2416, doi:10.18632/oncotarget.6262.
41. Zhang, Y.; Wang, H.; Ren, C.; Yu, H.; Fang, W.; Zhang, N.; Gao, S.; Hou, Q. Correlation Between C-MYC, BCL-2, and BCL-6 Protein Expression and Gene Translocation as Biomarkers in Diagnosis and Prognosis of Diffuse Large B-cell Lymphoma. *Frontiers in pharmacology* **2018**, *9*, 1497, doi:10.3389/fphar.2018.01497.
42. Wang, J.; Zhou, M.; Xu, J.-Y.; Chen, B.; Ouyang, J. Combination of BCL-2 and MYC protein expression improves high-risk stratification in diffuse large B-cell lymphoma. *Onco Targets Ther* **2015**, *8*, 2645-2650, doi:10.2147/OTT.S86093.
43. Nguyen, H.; Perry, A.; Skrabek, P.; Nasr, M.; Herrera, A.F.; Bedell, V.; Murata-Collins, J.; Pillai, R.; Xu, M.; Chen, L., et al. Validation of the Double-Hit Gene Expression Signature (DLBCL90) in an Independent Cohort of Patients with Diffuse Large B-Cell Lymphoma of Germinal Center Origin. *The Journal of molecular diagnostics : JMD* **2021**, *23*, 658-664, doi:10.1016/j.jmoldx.2021.02.005.
44. Isaksen, K.T.; Beiske, K.; Smeland, E.B.; Jørgensen, J.; Brodtkorb, M.; Myklebust, J.H.; Jerkeman, M.; Meriranta, L.; Karjalainen-Lindsberg, M.-L.; Leppä, S., et al. The DLBCL90 gene-expression assay identifies double-hit lymphomas with high sensitivity in patients from two phase II clinical trials with high-risk diffuse large B-cell lymphoma. *eJHaem* **2021**, *2*, 107-111, doi:10.1002/jha2.109.
45. Ennishi, D.; Jiang, A.; Boyle, M.; Collinge, B.; Grande, B.M.; Ben-Neriah, S.; Rushton, C.; Tang, J.; Thomas, N.; Slack, G.W., et al. Double-Hit Gene Expression Signature Defines a Distinct Subgroup of Germinal Center B-Cell-Like Diffuse Large B-Cell Lymphoma. *Journal of clinical oncology : official journal of the American Society of Clinical Oncology* **2019**, *37*, 190-201, doi:10.1200/jco.18.01583.
